# Let's stick together: Infection enhances preferences for social grouping in a songbird species

**DOI:** 10.1002/ece3.10627

**Published:** 2023-10-14

**Authors:** Marissa M. Langager, James S. Adelman, Dana M. Hawley

**Affiliations:** ^1^ Department of Biological Sciences Virginia Tech Blacksburg Virginia USA; ^2^ Department of Biological Sciences The University of Memphis Memphis Tennessee USA

**Keywords:** directly transmitted pathogen, host social preference, house finch, *Mycoplasma gallisepticum*, social behavior

## Abstract

Acute infections can alter foraging and movement behaviors relevant to sociality and pathogen spread. However, few studies have directly examined how acute infections caused by directly transmitted pathogens influence host social preferences. While infected hosts often express sickness behaviors (e.g., lethargy) that can reduce social associations with conspecifics, enhanced sociality during infection might be favored in some systems if social grouping improves host survival of infection. Directly assaying social preferences of infected hosts is needed to elucidate potential changes in social preferences that may act as a form of behavioral tolerance (defined as using behavior to minimize fitness costs of infection). We tested how infection alters sociality in juvenile house finches (*Haemorhous mexicanus*), which are both highly gregarious and particularly susceptible to infection by the bacterial pathogen *Mycoplasma gallisepticum* (MG). We inoculated 33 wild‐caught but captive‐held juvenile house finches with MG or media (sham control). At peak infection, birds were given a choice assay to assess preference for associating near a flock versus an empty cage. We then repeated this assay after all birds had recovered from infection. Infected birds were significantly more likely than controls to spend time associating with, and specifically foraging near, the flock. However, after infected birds had recovered from MG infection, there were no significant differences in the amount of time birds in each treatment spent with the flock. These results indicate augmented social preferences during active infection, potentially as a form of behavioral tolerance. Notably, infected birds showed strong social preferences regardless of variation in disease severity or pathogen loads, with 14/19 harboring high loads (5–6 log_10_ copies of MG) at the time of the assay. Overall, our results show that infection with a directly transmitted pathogen can augment social preferences, with important implications for MG spread in natural populations.

## Graphical abstract

Enhanced sociality during infection may benefit hosts if social grouping improves host survival of infection; however, little is known about how infected hosts may shift their social preferences during infection. We tested how infection with the bacterial pathogen *Mycoplasma gallisepticum* alters sociality in juvenile house finches by putting wild‐caught but captive‐held birds through a behavioral assay in which individuals were given the choice to associate near a flock or an empty cage. We found that infection with a directly transmitted pathogen can augment social preferences, with important implications for both pathogen spread and host survival in the wild.

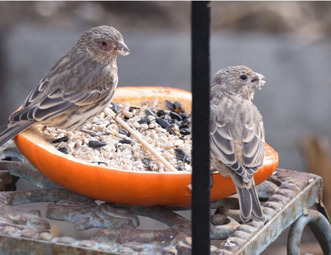

## 1 INTRODUCTION

Social interactions are critical for the spread of directly transmitted pathogens, yet infection often induces behavioral changes, such as sickness behaviors, that affect host sociality (Hawley et al., 2021; Stockmaier et al., 2021). Therefore, revealing how active infection alters host social preferences is important for understanding population‐level disease dynamics. Despite extensive work on how host sociality predicts transmission risk (e.g., Rifkin et al., 2012; Sah et al., 2018) and growing evidence that healthy hosts avoid infected conspecifics in many systems (e.g., Behringer et al., 2006; Poirotte et al., 2017; Stephenson, 2019), few studies specifically examine the social preferences of hosts actively infected with directly transmitted pathogens (Siva‐Jothy & Vale, 2019; Stephenson, 2019; Wu et al., 2023). Quantifying social preferences of infected hosts is critical because they can inform our understanding of important yet understudied host strategies for mitigating the fitness costs of infection, such as enhanced sociality for group‐living animals (Ezenwa et al., 2016).

Acute infections can alter host social preferences via diverse mechanisms, mediated by the pathogen or host. While some pathogens appear to manipulate infected hosts to increase sociality in ways that benefit pathogen transmission (Klein, 2003; Rode et al., 2013), the most common host‐mediated behavioral changes during infection are sickness behaviors (e.g., lethargy and anorexia (Hart, 1988)), which generally reduce social interactions and pathogen transmission potential (Cárdenas‐Canales et al., 2022; Hamilton et al., 2020; Lopes et al., 2016; Ripperger et al., 2020). However, social interactions may also be decreased when uninfected individuals actively avoid their infected conspecifics (Zylberberg et al., 2013), obscuring the true social preferences of infected hosts. Recent work suggests that gregariousness may reduce fitness costs of infection for hosts via improved food acquisition (Almberg et al., 2015; Ezenwa & Worsley‐Tonks, 2018), territory defense (Almberg et al., 2015), and increased predator vigilance by conspecifics (Ezenwa & Worsley‐Tonks, 2018). Thus, sociality during infection may act as a key form of “behavioral tolerance” by improving host survival of infection (Ezenwa et al., 2016; Stockmaier et al., 2023). Direct assays of social preferences of actively infected hosts are crucial for revealing how hosts cope with infection behaviorally, and the potential consequences of these responses for pathogen spread.

**FIGURE 1:**
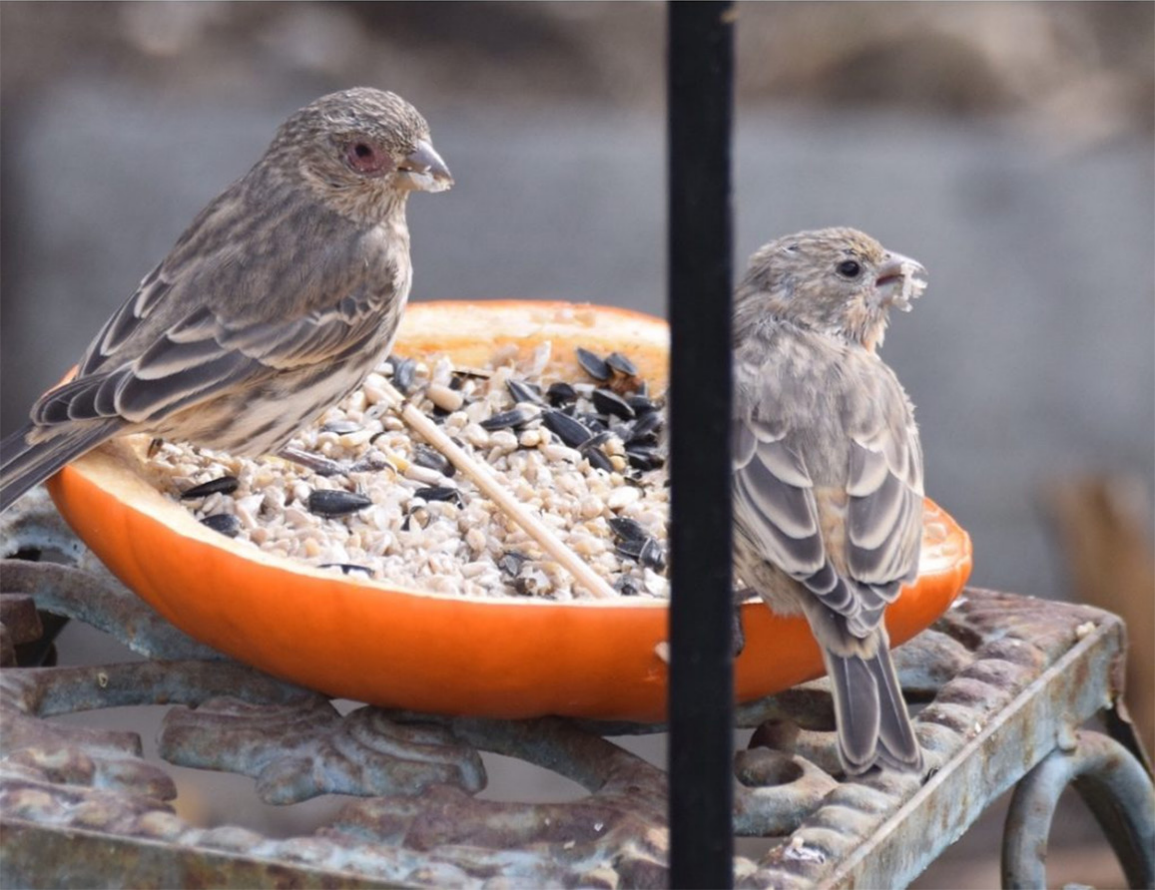
Two juvenile house finches eating together at a bird feeder. The bird on the left has noticeable clinical signs of *Mycoplasma gallisepticum* (MG) infection (redness and swelling of the conjunctiva). In contrast, the bird on the right shows no signs of MG infection. Photograph taken by Ivey Fennell, access for use courtesy of the Cornell Lab of Ornithology Project FeederWatch.

We tested how experimental infection influences social preferences in a naturally occurring host–pathogen system, house finches (*Haemorhous mexicanus*) and the bacterial pathogen *Mycoplasma gallisepticum* (MG), which causes conjunctivitis in this species (Kollias et al., 2004; Figure 1). House finches are gregarious songbirds that commonly experience MG outbreaks during the nonbreeding season, when flocks congregate to forage at bird feeders (Hosseini et al., 2009). Feeders facilitate MG spread through shared use of fomites and augmentation of direct contacts between conspecifics (Adelman et al., 2015; Dhondt et al., 2007; Figure 1). Because MG has a short survival time on feeder surfaces (Dhondt et al., 2007) and MG prevalence is density dependent (Altizer, Hochachka, et al., 2004), social preferences of infected birds at feeders are likely critical for transmission. This may be particularly true for juvenile hatch‐year birds, which join large foraging flocks and harbor high MG prevalence (Altizer, Davis, et al., 2004), suggesting they are important drivers of MG epidemics (Hosseini et al., 2009).

Behavioral studies show that MG infection causes sickness behaviors including lethargy (Kollias et al., 2004) and reduced behavioral responses to visual predator stimuli (Adelman et al., 2017). While the conjunctivitis associated with MG infection can be sufficiently severe to obscure vision (Kollias et al., 2004), infected house finches show behavioral changes such as reduced antipredator responses even in the absence of severe eye swelling (Adelman et al., 2017). With respect to social behaviors, free‐living finches with conjunctivitis are observed in smaller flocks than those of healthy birds (Hawley et al., 2007; Hotchkiss et al., 2005). Because uninfected finches do not avoid MG‐infected conspecifics (Bouwman & Hawley, 2010), such patterns may reflect decreased sociality of actively infected hosts, a common component of sickness behaviors. However, these patterns could also reflect an inability of diseased birds to move readily among feeding sites (Hawley et al., 2007), rather than social preferences. In fact, infected finches may directly benefit from social behaviors because MG reduces antipredator behaviors in house finches (Adelman et al., 2017), a source of MG‐mediated mortality that may be partially offset by flock membership during infection (Cresswell, 1994). Overall, while past studies document how the behaviors of individually housed birds change during MG infection (Kollias et al., 2004) and whether healthy house finches avoid MG‐infected flockmates (Bouwman & Hawley, 2010), the social preferences of infected birds have not yet been directly examined. Understanding how social preferences toward healthy conspecifics change during acute infection, and whether such changes occur in ways that might benefit infected hosts or influence ongoing transmission, requires assays that explicitly quantify the social preferences of infected hosts.

The house finch‐MG system offers an opportunity to directly test whether infected hosts show decreased sociality due to sickness behaviors, increased sociality as a potential form of behavioral tolerance, or neither. Further, because there is individual variation in disease severity in response to MG infection in house finches (Adelman et al., 2017), this system also provides important insights into how the social preferences of birds with less severe disease and overall lethargy may influence disease dynamics in this system. To elucidate whether and how MG infection influences social preferences, we experimentally inoculated hatch‐year house finches with MG or control media and used choice assays to compare social preferences of infected versus uninfected individuals. We also examined whether heterogeneity in infection severity predicts variation in sociality, which would potentially underlie individual‐level covariation in infectiousness and contact rates (Stephenson, 2019). Finally, to investigate whether any detected changes in social preferences were related to active infection per se, we conducted this same choice assay after infected birds were allowed to recover.

## 2 METHODS

### 2.1 Study subjects, sexing, and housing

Thirty‐three hatch‐year house finches, used as *focal birds* (20 males, 13 females; 1–3 months old), were captured in Blacksburg, Virginia, USA and the City of Radford, Virginia, USA in May and June 2019. Three of these birds were collected as nestlings and hand‐fed until nutritional independence (their inclusion did not alter results; see Section 3); the remaining 30 were nutritionally independent at capture. Age (hatch‐year or after hatch‐year) was determined at capture by plumage, lack of a brood patch or cloacal protuberance, and the presence of a distinct yellow gape line. All birds showed no clinical signs of MG infection, and all birds were seronegative for prior MG exposure (Hawley et al., 2011) prior to experimental infection. Sex was assigned to each bird prior to the start of the experiment using DNA extracted from packed red blood cells using DNeasy 96 Blood and Tissue Kit (Qiagen). The presence of sex chromosomes (ZW for females and ZZ for males) was determined using PCR (Griffiths et al., 1998).

Upon capture, all birds were housed in pairs in cages (76 × 46 × 46 cm) for up to a month depending on capture date. All birds were kept in indoor temperature‐controlled rooms with a 12L:12D light cycle for the duration of the study. All birds were moved into individual cages of the same size 1 week before inoculation, where they were housed for the remainder of the experiment.

### 2.2 Stimulus birds

Eight additional hatch‐year house finches served as our flock *stimulus birds* for assaying social preferences. All stimulus birds showed no clinical signs of MG infection and were all seronegative for prior MG exposure (Hawley et al., 2011) before use in the behavioral assays. Stimulus birds were housed in separate rooms from all focal birds (prior to behavioral assays) to keep focal individuals unacquainted with the stimulus flock. Further, even during behavioral assays, stimulus birds remained in separate cages from focal birds, preventing any MG transmission to stimulus birds. Four days prior to the start of behavioral assays, four of the eight stimulus birds were placed together into a new cage in the room where the sociality assay occurred. The first group of four stimulus birds were used for 40 trials (two replicate trials for 20 unique focal birds). After 40 trials, these four stimulus birds were switched out with a different flock of four birds, which were used as the stimulus birds for the remaining 26 behavioral trials (two replicate trials for 13 unique focal birds).

### 2.3 Inoculation and behavioral assays

Focal birds were randomly assigned to treatment using a random number generator within sex, with higher sample sizes allotted to the infection versus control treatment to account for heterogenous responses to infection (MG infection treatment: *n* = 19; sham control treatment: *n* = 14). Birds were split into two experimental rounds (7 days apart; each individual bird was only included in one unique round) in order to complete all behavioral assays during the infectious period (Days 10–20 post infection (Dhondt et al., 2008)), when sociality is most relevant for ongoing spread. On experimental Day 0, birds were inoculated bilaterally in the conjunctiva with 35 μL of MG (infection treatment) in Frey's media or with media alone (sham control treatment). We used an MG strain collected in North Carolina, USA, in 2006 (NC2006, 2006.080–5 4P 7/26/12, David H. Ley, NC State University, College of Veterinary Medicine, Raleigh, NC, USA 27606), with a viable count of 2.49 × 10^6^ color‐changing units (CCUs).

We monitored disease severity weekly and on the day of behavioral assays by scoring conjunctivitis on a 0–3 scale per side, with scores of 3 representing severe conjunctivitis (Hawley et al., 2011). Scores for each side (left and right) were summed within sampling day for a maximum total eye score of 6 for a given focal bird. We swabbed conjunctiva weekly post inoculation to quantify MG load, as well as immediately after behavioral trials if weekly swabs did not fall within ±2 days of a given bird's behavioral assay. Swabs were stored in 300 μL of tryptose phosphate broth (TPB) and stored at −20°C until extraction using Qiagen 96 DNeasy Blood and Tissue Kit; the amount of MG in each sample was determined via a probe‐based qPCR using methods outlined in prior work (Hawley et al., 2011).

**FIGURE 2:**
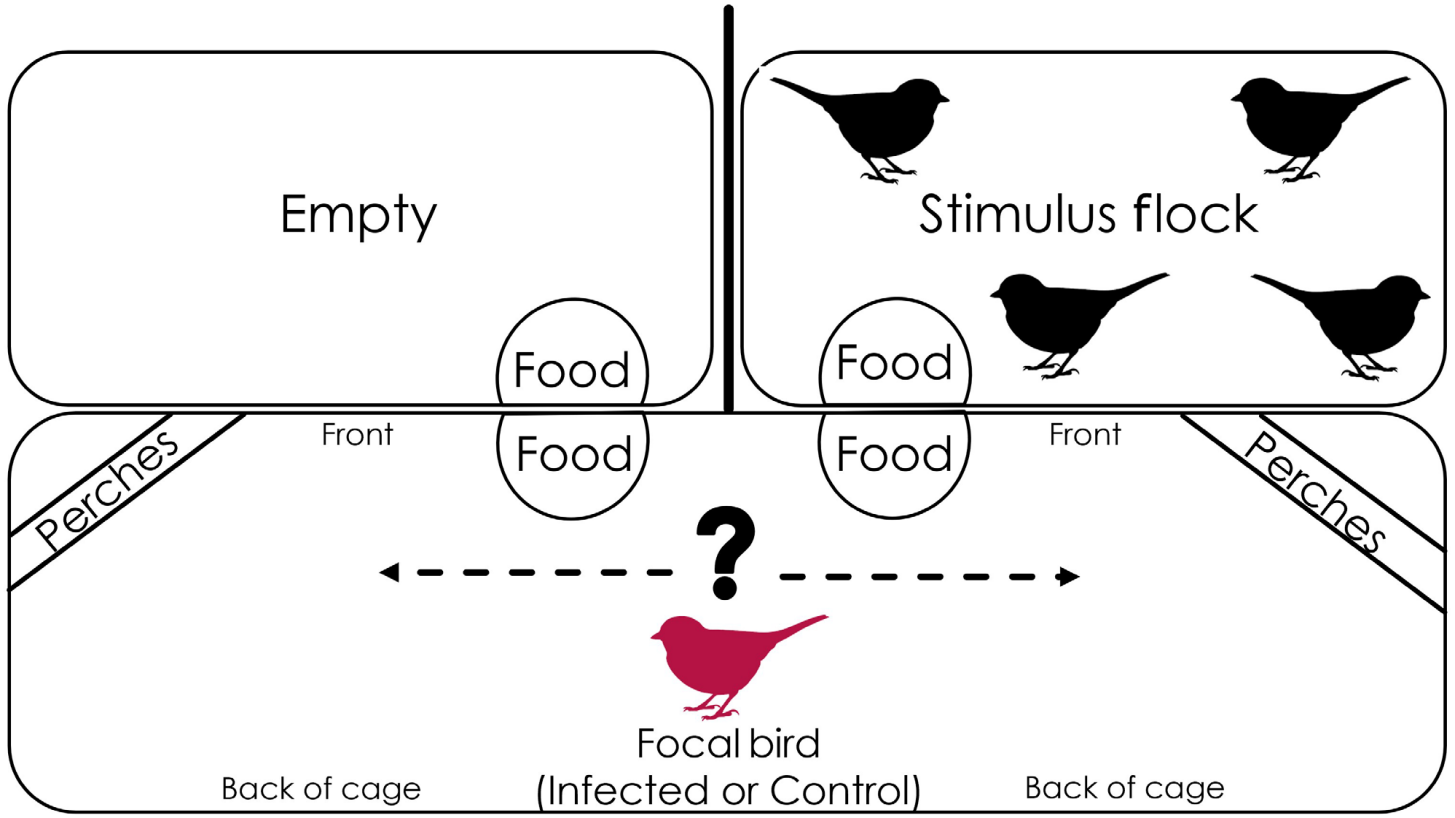
Top‐down view of social preference behavioral arena, with food dishes at the front of the focal cage (dimensions: 105 × 46 × 40 cm). This large focal cage was placed directly in front of two smaller stimulus cages (dimensions: 76 × 46 × 46) containing a flock of four juvenile stimulus birds. The side of the stimulus flock was switched between replicates for a given focal bird such that every focal bird was assayed with the stimulus flock on each side.

Each focal bird was tested on two consecutive days within their peak infectious period (post infection Days 10–20 (Dhondt et al., 2008)) and all behavioral assays occurred between 07:30 and 10:50, and food was withheld from focal birds for 3 hours before testing to standardize motivation. Focal birds were placed in a behavioral arena (Figure 2) where they could feed in proximity to a stimulus cage containing four unfamiliar, uninfected conspecifics on one side, or an empty cage on the other, and video recorded for 45 min. To account for side preferences unrelated to the presence of stimulus birds, we repeated the assay for each focal individual on consecutive mornings: once with the stimulus flock on each side of the cage (order was randomized). We quantified preference by recording time spent in one of two mutually exclusive behaviors (perching or eating) on each side of the arena during 35 min per replicate assay (allowing 10 min for acclimation). Videos were split randomly between two observers so that each observer watched videos from both infected and control individuals, while always remaining blind to treatment. However, both of an individual bird's trials were observed by the same individual.

Thirty‐one days after inoculation, infected birds were given a broad‐spectrum antibiotic (Tylan®, tylosin tartrate) in their drinking water (at a concentration of 1 g/L water) for 5 weeks until all birds showed no clinical signs of MG. After all birds were recovered from infection, we repeated the choice assay with eight new stimulus birds. The first group of four stimulus birds were used for 38 trials (two replicate trials for 19 unique focal birds). After 38 trials, the other group of four stimulus birds were used for the remaining 26 behavioral trials (two replicate trials for 13 unique focal birds). All postinfection videos were watched and coded using BORIS (Friard & Gamba, 2016).

### 2.4 Statistical analyses

All data were analyzed in R v 3.6.1 (R Core Team, 2021). For both of our assays (during infection and post infection), we calculated two behavioral metrics: (1) the proportion of time a focal bird spent perching near the stimulus flock and (2) the proportion of time spent eating near the stimulus flock (with eating defined as a bird being perched on the food dish and pecking at food at least every 20 s). Our definition of each behavior resulted in the time spent in each behavior as mutually exclusive (i.e., a bird perched on the food dish and actively pecking at food was designated as “eating” but not “perching”). Thus, we also calculated a summary measure of preference to associate with the flock as the proportion of time each bird spent either perching, eating, or both perching and eating near the stimulus flock. For each variable, we summed a bird's time engaged in that activity (eating, perching, or either) near the stimulus flock across replicate trials (for 70 total minutes of observation), utilizing only data from the front half of the arena (near the stimuli), which represented >98% of assay time. We then divided these sums by the total time spent engaged in the respective activity (eating, perching, or either). Thus, although each bird in our study had two replicate trials (with the stimulus flock located on each side of the arena), only one response value per behavior was analyzed for each unique focal bird in our study. Three infected birds did not eat during the infection assay, consistent with prior work documenting infection‐induced anorexia in this species (Adelman et al., 2013); thus, these three birds were only included in the perching model and the combined model of eating or perching. One bird died prior to starting our post infection assays, so only 32 birds of the original 33 birds were tested once infected birds had recovered.

We used these proportions as response variables in separate generalized linear models (GLMs, using quasibinomial error distributions) with treatment (infected or control; or recovered or control for post recovery assays) as the main effect. Models were weighted by total time eating (eating model), total time perching (perching model), or total time engaged in either behavior (combined perching or eating model). We tested for significance using *t*‐values generated by our GLM for each variable in R. Sex, day post infection (which always fell between Days 10 and 20 but varied across individuals), and experimental round were initially included in all infection models, but covariates were removed from final models if the GLM parameter estimate for that covariate and associated *t*‐test was *p* > .1. Only sex and experimental round were included as covariates in our post infection models and were also removed from the final model using the cutoff stated above. Within the infected treatment only, we also asked whether variation in the severity of conjunctivitis or pathogen load at the time of the sociality assay predicted behavioral preference. We used ggplot2 (Wickham, 2016) for all graphing.

## 3 RESULTS

**FIGURE 3:**
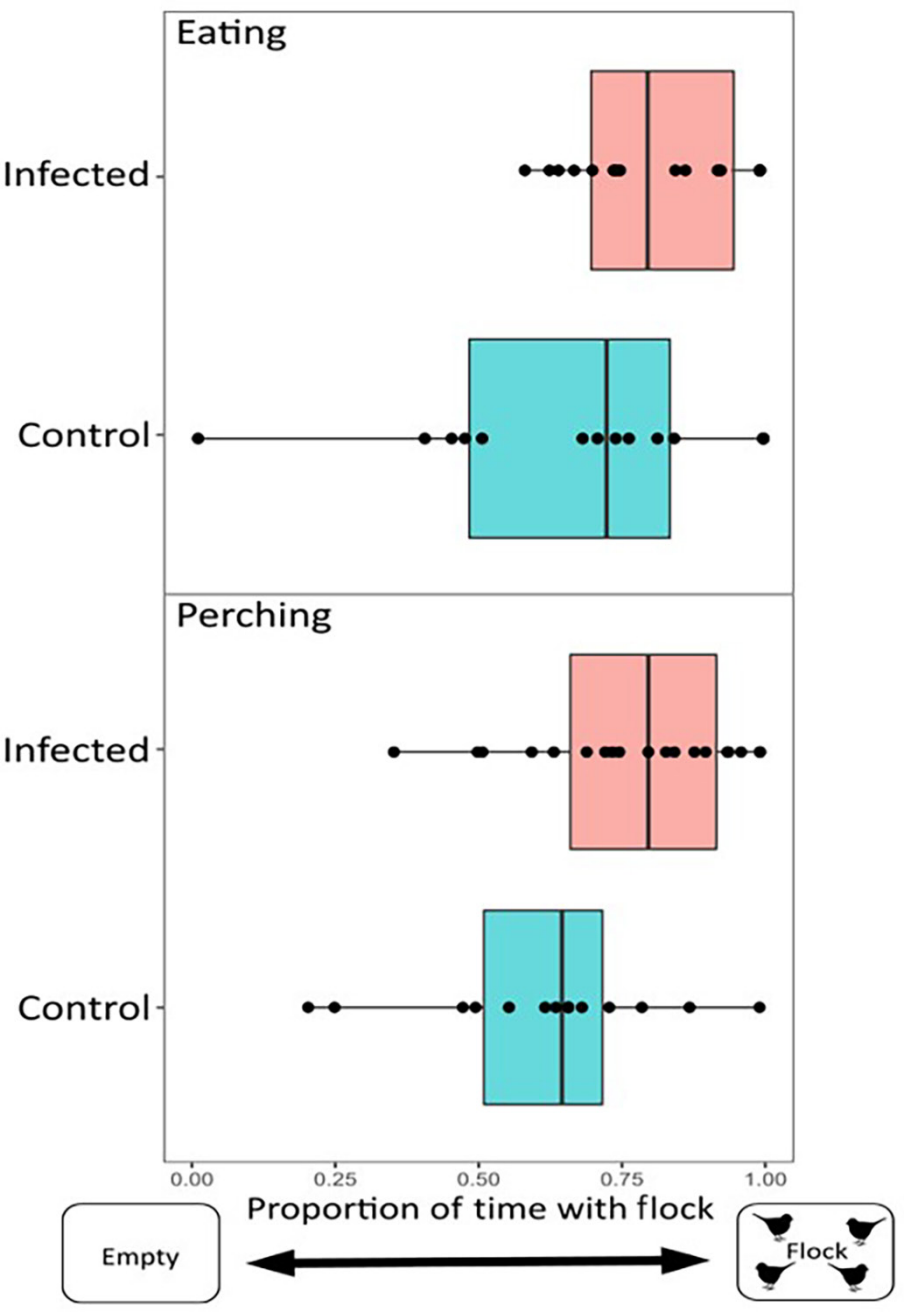
House finches infected with *Mycoplasma gallisepticum* spent significantly more time eating (*p* = .018; *n* = 16 individuals) though not significantly more time perching (*p* = .062; *n* = 19 individuals), near a flock of novel conspecifics than did uninfected controls (*n* = 14 individuals). Note that the sample sizes are lower for time eating versus perching because three infected individuals did not eat during the assay (see Section 2).

**FIGURE A1:**
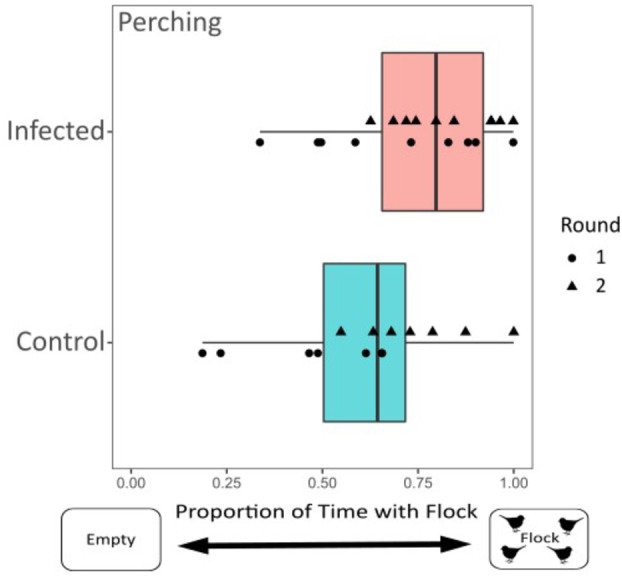
For all birds, experimental round (Round 1, circles; Round 2, triangles) was a significant covariate in the generalized linear models for perching only. Any effect of round was accounted for in our analysis and, thus, did not influence our interpretation of treatment effects.

For our behavioral trials performed during infection, there was individual variation within and between treatments in time spent eating (infected: 1.47–46.27 min; control: 0–34.01 min) and perching (infected: 15.93–59.88 min; control: 2.23–62.56 min) near the flock, out of an average total assay time of 70 min (2 replicates of 35 min each). For eating, this variation was significantly predicted by infection treatment, with infected house finches spending significantly more time eating near the stimulus flock, relative to uninfected birds (Figure 3; *n* = 30; Intercept (Control) = 0.55 ± 0.24, Beta (Infected) = 1.07 ± 0.43, *t* = 2.51, *p* = .018). However, we did not find statistically significant support for effects of infection treatment on time perching near the stimulus flock (Figure 3; *n* = 33; Intercept (Control) = −0.92 ± 0.52, Beta (Infected) = 0.62 ± 0.32, *t* = 1.94, *p* = .062). When the two quantified behaviors were pooled in a combined analysis (time spent eating or perching with the flock), infected house finches were significantly more likely to spend time associating with the flock when engaged in either behavior (*n* = 33; Intercept (Control) = −0.56 ± 0.49, Beta (Infected) = 0.69 ± 0.30, *t* = 2.30, *p* = .028), relative to uninfected individuals. All covariates included in initial models (see Section 2) showed *p* > .1 and were removed, except experimental round in the model of perching (Beta (Round 2) = 0.95 ± 0.32, *t* = 2.97, *p* = .01) and the combined model of time spent eating or perching (Beta (Round 2) = 0.72 ± 0.30, *t* = 2.39, *p* = .02) (Figure A1).

**FIGURE 4:**
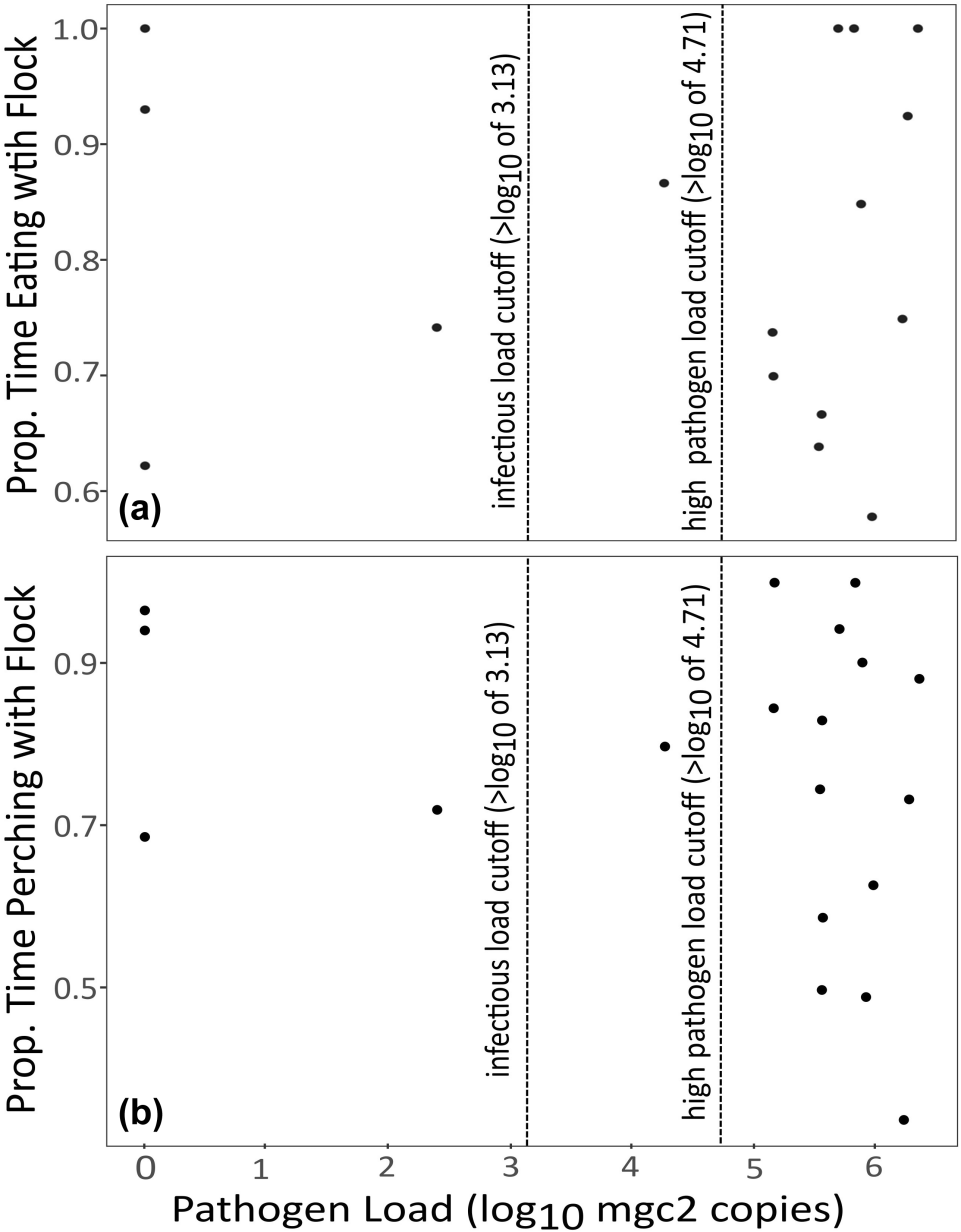
Among infected birds, there was no significant relationship between individual variation in pathogen load at the time of assay (*x*‐axis) and the proportion of time eating (Panel a; *n* = 16) or perching (Panel b; *n* = 19) near the stimulus flock (*y*‐axis). At the time of assay, infected house finches largely had conjunctival pathogen loads that were above the infectious load for MG (Adelman et al., 2015) (loads ≥3.13 log_10_ copies of MG; 15/19 birds; left vertical dashed line). We further defined pathogen loads as “high” if they fell above the average pathogen load for the NC2006 isolate detected in a past study (Fleming‐Davies et al., 2018) (loads ≥4.71 log_10_ copies of MG; right vertical dashed line), which was the case for 14/19 infected birds at the time of assay.

Birds in the infected treatment showed variable disease severity at the time of assay, from summed (left plus right conjunctiva) severity scores of 0.5 to 6 (mean: 3.76, SD: 1.91) out of a maximum of 6. However, among infected birds, severity of conjunctivitis did not predict the proportion of time eating (*n* = 16; Intercept = 1.38 ± 0.52, Beta = 0.07 ± 0.14, *t* = −0.53, *p* = .60), perching (*n* = 19; Intercept = 1.78 ± 0.58, Beta = −0.18 ± 0.13, *t* = −1.38, *p* = .18), or generally associating (eating or perching) with the flock (*n* = 19; Intercept = 1.70 ± 0.51, Beta = −0.13 ± 0.12, *t* = −1.15, *p* = .27). Pathogen load in the conjunctiva at the time of assay varied from 0 to 6.35 log_10_ copies of MG (mean: 4.59 log_10_ copies of MG, SD: 2.22 log_10_ copies of MG) for infected birds, with 14/19 birds harboring “high” MG loads (defined as ≥ 4.71 log_10_ copies of MG, the average load for this isolate (Fleming‐Davies et al., 2018)) and 15/19 harboring loads predicted to be infectious (defined as ≥ 3.13 log_10_ copies of MG as per (Adelman et al., 2015)). Among infected birds, pathogen load did not predict the proportion of time spent eating (Figure 4a; *n* = 16; Intercept = 2.26 ± 0.64, Beta = −0.14 ± 0.12, *t* = −1.14, *p* = .27), perching (Figure 4b; *n* = 19; Intercept = 1.88 ± 0.67, Beta = −0.17 ± 0.13, *t* = −1.30, *p* = .21), or generally associating (eating or perching) with the flock (*n* = 19; Intercept = 2.0 ± 0.60, Beta = −0.17 ± 0.11, *t* = −1.49, *p* = .16).

**FIGURE 5:**
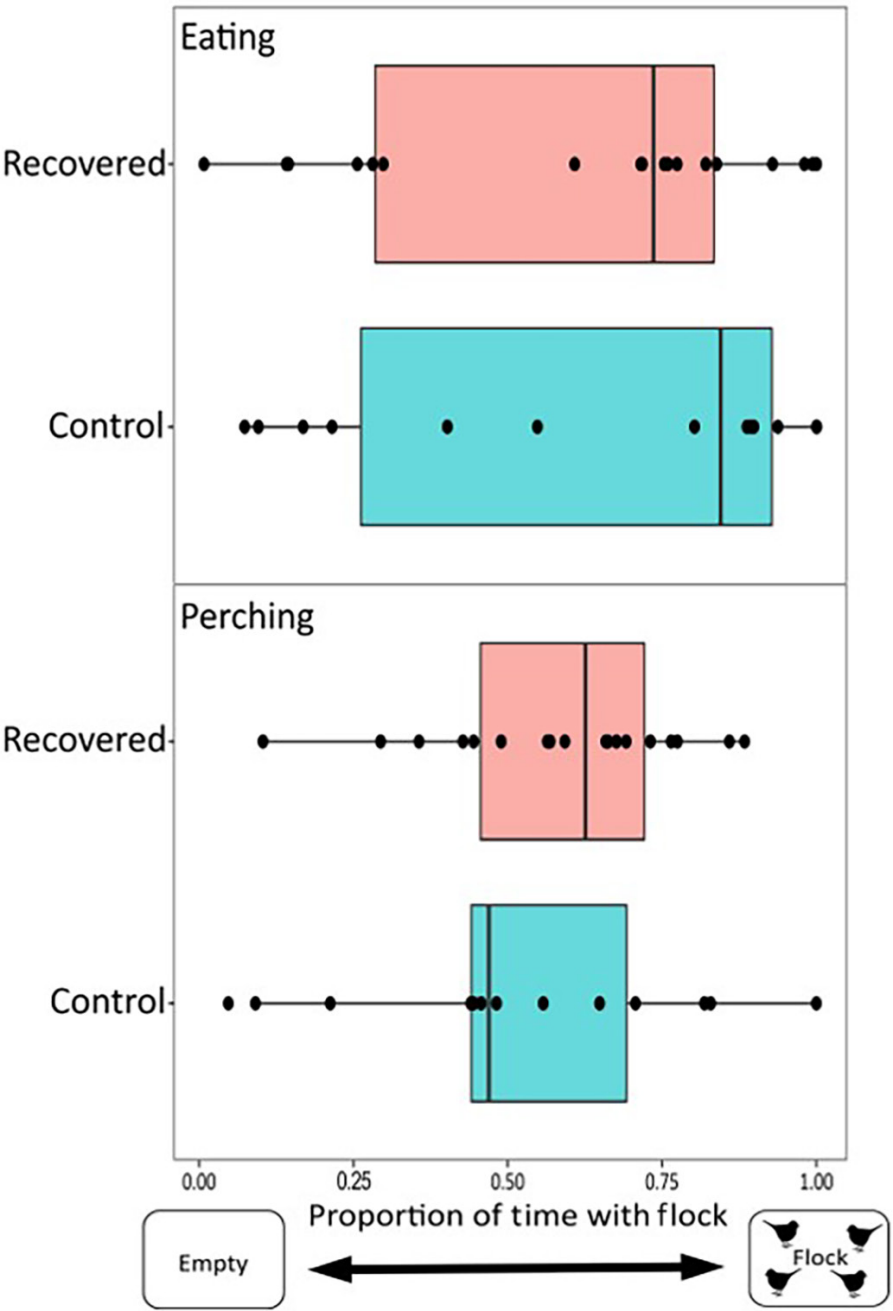
House finches that had recovered from *Mycoplasma gallisepticum* did not spend a significant amount of time eating (*p* = .28, *n* = 18 individuals) or perching (*p* = .12, *n* = 18 individuals) near a flock of novel conspecifics than did uninfected controls (*n* = 14 individuals).

For our behavioral trials performed after infected birds had recovered, there was also individual variation within treatment in the amount of time spent eating (recovered: 0–49.63 min; control: 1.2–61.78 min) and perching (recovered: 3.58–38.82 min; control: 1.73–35.87 min) near the flock, out of an average total assay time of 72 min (2 replicates of 36 min each). However, in contrast to assays during active infection, a bird's prior infection treatment (recovered or uninfected control) did not significantly predict either the amount of time eating near the stimulus flock (Figure 5; *n* = 32; Intercept (Control) = −1.35 ± 0.84, Beta (Infected) = −0.60 ± 0.54, *t* = −1.11, *p* = .28) or the amount of time spent perching near the flock (Figure 5; *n* = 32; Intercept (Control) = −0.16 ± 0.25, Beta (Infected) = 0.56 ± 0.35, *t* = 1.58, *p* = .12). When eating and perching behaviors were pooled, there was no significant difference between treatments in the amount of time spent associating with the flock (*n* = 32; Intercept (Control) = −1.15 ± 0.69, Beta (Infected) = 0.02 ± 0.42, *t* = 0.04, *p* = .97) In all post‐recovery models, covariates were removed if they showed *p* > .1, with the exception of experimental round in our eating model (Beta (Round 2) = 1.52 ± 0.54, *t* = 2.82, *p* = .01) and the combined model (Beta (Round 2) = 0.97 ± 0.42, *t* = 2.31, *p* = .03).

To ensure that the inclusion of three hand‐fed birds did not alter our results, we repeated the GLMs (using quasibinomial error distributions) with these birds excluded from the analysis. We found that there were no differences in the effects of treatment on the amount of time spent eating (*n* = 27; Intercept (Control) = 0.524 ± 0.249, Beta (Infected) = 1.25 ± 0.480, *t* = 2.60, *p* = .015) or perching (*n* = 30; Intercept (Control) = 0.459 ± 0.279, Beta (Infected) = 0.6684 ± 0.369, *t* = 1.81, *p* = .082) near the flock during infection compared to the models including these three hand‐fed birds.

## 4 DISCUSSION

We found that house finches actively infected with a directly transmitted pathogen spent significantly more time than uninfected controls associating with, and specifically eating near, a flock of healthy conspecifics. Notably, birds in the infected treatment generally displayed uniformly high levels of sociality, regardless of individual variation in their disease severity or pathogen load at the time of assay. Because most (15/19) infected birds harbored pathogen loads well above prior estimates for MG's minimum infectious dose in finches (Adelman et al., 2015), such augmented sociality likely has key consequences for transmission. In this system, pathogen transmission increases with both the time that birds spend on feeders (Adelman et al., 2015) and the degree of host pathology (Bonneaud et al., 2020; Ruden & Adelman, 2021), which enhances pathogen deposition onto bird feeders (Adelman et al., 2013). Because finches with severe pathology are often less active (Adelman et al., 2017), pathogen spread is predicted to be maximized at moderate degrees of conjunctivitis severity (Bonneaud et al., 2020). Thus, the augmented sociality seen during infection here, including in finches with high pathogen loads (Figure 4) but only moderate pathology (e.g., 25th–75th percentiles, or Scores 2–5 in this study, *n* = 9/19 birds), is likely to facilitate MG spread in the wild.

Changes in behavior during infection can broadly be driven by host‐ or pathogen‐mediated mechanisms, including direct manipulation of host behavior by pathogens. Directly transmitted parasites should benefit from manipulating host sociality, and some studies show higher sociality in infected animals consistent with parasite manipulation of host behavior (Petkova et al., 2018; Rode et al., 2013). Nonetheless, examples of parasite manipulation to increase host sociality are rare, with observed behavioral changes more often manifesting as host‐mediated declines in sociality (Cárdenas‐Canales et al., 2022; Hawley et al., 2021). Our results represent a case of a directly transmitted pathogen causing augmented rather than reduced host sociality, potentially due to host‐mediated behavioral changes. While our experimental design does not allow us to rule out the possibility that the observed behavioral changes are pathogen‐mediated, Poulin (2010) hypothesized that selection on directly transmitted parasites to manipulate the sociality of gregarious hosts is rare because such parasites already have ample transmission opportunities. Further, in systems where augmented sociality during infection has been observed, there are clear hypothesized benefits to hosts for such behavioral changes. For example, Stephenson (2019) found increases in sociality in male guppies (*Poecilia reticulata*) that harbored the highest loads of a directly transmitted ectoparasite, a behavioral change that the authors hypothesized may increase mating opportunities and the ability to permanently shed worms onto other hosts, potentially benefiting infected host fitness. Further, Wu et al. (2023) found that *C. elegans* hermaphrodites will shift their mating preferences when exposed to a bacterial pathogen, increasing the rate that they associate and mate with males. Together with our results, such studies indicate that infected hosts in some systems augment sociality in ways that likely ultimately benefit host fitness. However, it is notoriously challenging to tease apart whether behavioral changes during infection represent host‐mediated changes, pathogen‐mediated changes, or some combination (Nadler et al., 2023).

Due to the energetic costs of both MG infection and social behaviors, as well as the lethargy common among house finches infected with MG (Kollias et al., 2004), increased sociality during infection may seem counterintuitive as a potential host‐mediated strategy. However, maintenance of social behaviors may be one form of behavioral tolerance in this system, lowering the survival costs of infection (Ezenwa et al., 2016). One cost of MG infection in house finches is a reduction in antipredator behaviors (Adelman et al., 2017), which likely contributes to MG‐related mortality in the wild (Faustino et al., 2004). Birds that forage with flocks while infected would likely have increased protection from predation threats (Fernández‐Juricic et al., 2004), and thus higher likelihood of surviving infection. However, it must be noted that, given the reduced ability of infected finches to evade capture in mock predation trials (Adelman et al., 2017), associating with flocks may also elevate predation risk for infected birds if larger flocks attract more predators and infected birds serve as easier targets than their uninfected flockmates. Interestingly, differences in sociality between infected individuals and uninfected controls were no longer present once infected birds had recovered from infection, which may further indicate that infected birds utilize increased sociality to offset the costs of sickness behavior, which becomes unnecessary after recovery.

Another mechanism that may alleviate the high fitness costs of infection is improved foraging and food acquisition (Ezenwa et al., 2016; Ezenwa & Worsley‐Tonks, 2018), a key benefit of flocking behavior in nonbreeding birds (Fernández‐Juricic et al., 2004). During infection, sickness behaviors like lethargy may decrease an individual's ability to locate or use a food source (Ezenwa et al., 2016). Group membership may offset these foraging costs of sickness behavior by assisting infected individuals in locating or acquiring a food source (Almberg et al., 2015) or through increased predator vigilance, allowing infected animals to allocate more time toward foraging (Ezenwa & Worsley‐Tonks, 2018). Given that infected birds were significantly more likely to associate with the flock while eating but not perching in our study, foraging benefits of sociality may be particularly important during infection. Notably, even control birds showed a nonrandom preference to feed near the flock versus the empty cage, though that preference was not as strong as that seen in infected birds. This likely reflects the benefits of group feeding in this species and their high degree of sociality (Badyaev et al., 2020). Although the hypothesized effects of MG infection on perching behavior, which included any resting or preening behaviors done while remaining perched in one location in the arena, did not have statistically significant support, the detected patterns for perching behavior in infected versus control birds were qualitatively similar to that found for time eating (Figure 3). When the two behaviors were pooled, this contributed to an overall significant preference for infected birds to associate with the flock when either eating or perching in our combined analysis. Overall, the potential anti‐predation and foraging benefits of sociality are likely not mutually exclusive in house finches, with social groups providing multiple benefits to infected individuals.

The preferences for augmented sociality seen in infected birds in our study could also reflect changes in the relative cost–benefit ratio associated with sociality. For example, while increased risk of infection is considered a broader cost of sociality (Hawley et al., 2021), already infected hosts may be less motivated to avoid this cost. In a study of avoidance of infected conspecifics in a gregarious lobster species, Caribbean spiny lobsters (*Panulirus argus*) were given a choice to den alone or with a virus‐infected conspecific; while healthy lobsters strongly avoided denning with an infected conspecific, infected lobsters showed no detectable preference (Behringer et al., 2006). Enhanced social preference of infected birds could also result from more generalized, and potentially non‐adaptive, changes to host sensory processing whereby infected birds are attracted to feed near a wide range of sensory stimuli; however, prior work showing that infected house finches are less responsive than healthy birds to both visual and auditory stimuli of potential predation threats (Adelman et al., 2017) suggests that generalized attraction is unlikely in this system. Further research should examine whether the social preferences seen in infected versus uninfected birds in our study result from potential benefits of sociality to infected birds (e.g., reduced predation risk, increased foraging efficiency), reduction in the potential costs of sociality for infected birds (e.g., increased infection risk), changes in generalized attraction to sensory stimuli during infection, or some combination thereof. Interestingly, house finches from populations that have had longer time with MG endemic in their population display lower conjunctivitis severity per unit pathogen (Henschen et al., 2023), suggesting that natural populations that have coevolved with MG show potential adaptive responses to MG infection. Performing MG inoculations of birds from populations where MG has not yet been documented may help to elucidate whether the behavioral changes detected here represent evolved strategies of behavioral tolerance to MG infection, though such differences may have evolved in response to infection and sickness behaviors more generally. Finally, we cannot eliminate the possibility that pathogen‐mediated manipulation contributes to the augmented sociality in infected house finches, which could be assessed using noninfectious immune challenges.

Regardless of the mechanisms driving our results, the increased time that infected birds spend eating near conspecifics is likely to have important consequences for MG transmission. This pathogen appears to spread primarily at bird feeders (Adelman et al., 2015) from indirect contacts that occur within minutes to hours, when MG deposited onto surfaces from infected birds is still viable (Dhondt et al., 2007). Increases in the probability that infected birds feed in the presence of a flock should therefore enhance fomite‐based transmission. Thus, uninfected birds in flocks might be expected to actively avoid eating near their infected conspecifics, regardless of the infected individual's social preferences. However, it has been found that uninfected house finches do not actively avoid eating near MG‐infected individuals, and in some cases male house finches preferentially feed near infected versus healthy male conspecifics (Bouwman & Hawley, 2010). While no studies have specifically looked at the mechanisms driving the lack of avoidance of infected conspecifics in this system, such behaviors may arise because the benefits of flocking behavior in this system outweigh the costs, even for uninfected individuals. Overall, because uninfected birds do not actively avoid infected conspecifics (Bouwman & Hawley, 2010), our findings on the social preferences of the infected flockmates are especially interesting and suggest that augmented sociality plays a key role in determining disease dynamics within this system.

While our behavioral assays allowed us to specifically isolate social preferences of infected versus uninfected birds, these assays also have limitations when extrapolating to social behaviors and transmission implications in the wild. The captive behavioral arena may not reflect the energetic costs an infected bird incurs while moving with flocks of uninfected conspecifics. In our small arena, even birds with the most severe pathology were able to move and eat without utilizing much energy, an unlikely situation in wild flocks. This may explain why we found no relationship between individual variation in disease severity and time spent associating near the flock in our assays. While our experiment showed that infected birds almost universally prefer to forage near a flock, only individuals with low to moderate pathology may be able to exercise their social preferences in the wild by keeping up with mobile foraging flocks (Hawley et al., 2007). Overall, future attention should be put on the implications of these preferences for transmission in the wild, focusing on whether only those animals with moderate pathology are able to carry out their social preferences and, thus, become primary drivers of pathogen transmission across a landscape.

## AUTHOR CONTRIBUTIONS

**Marissa M. Langager:** Conceptualization (lead); data curation (lead); formal analysis (lead); investigation (lead); methodology (lead); project administration (lead); visualization (lead); writing – original draft (lead); writing – review and editing (lead). **James S. Adelman:** Formal analysis (supporting); funding acquisition (lead); writing – original draft (supporting); writing – review and editing (supporting). **Dana M. Hawley:** Conceptualization (supporting); funding acquisition (lead); investigation (supporting); methodology (supporting); resources (lead); visualization (supporting); writing – original draft (supporting); writing – review and editing (supporting).

## CONFLICT OF INTEREST STATEMENT

The authors declare no competing financial interests.

## Data Availability

Data and R code for the study and analyses are deposited in the open access Virginia Tech Data Repository at https://doi.org/10.7294/19522195.
